# Immune landscape and heterogeneity of cervical squamous cell carcinoma and adenocarcinoma

**DOI:** 10.18632/aging.205397

**Published:** 2024-01-10

**Authors:** Binghan Liu, Yashi Xu, Bai Hu, Xiaole Song, Shitong Lin, Jiaxuan Wang, Lingfang Wang, Tian Chu, Ting Peng, Miaochun Xu, Wencheng Ding, Canhui Cao, Peng Wu, Li Li

**Affiliations:** 1 Department of Obstetrics and Gynecology, Union Hospital, Tongji Medical College, Huazhong University of Science and Technology, Wuhan 430022, Hubei, China; 2 Department of Gynecologic Oncology, Tongji Hospital, Tongji Medical College, Huazhong University of Science and Technology, Wuhan 430030, Hubei, China; 3 Cancer Biology Research Center (Key Laboratory of The Ministry of Education), Tongji Hospital, Tongji Medical College, Huazhong University of Science and Technology, Wuhan 430100, Hubei, China; 4 Department of Gynecologic Oncology, The First Affiliated Hospital of Zhengzhou University, Zhengzhou 450052, Henan, China; 5 BGI-Shenzhen, Shenzhen 518083, Guangzhou, China; 6 Zhejiang Provincial Key Laboratory of Precision Diagnosis and Therapy for Major Gynecological Diseases, Women's Hospital, Zhejiang University School of Medicine, Hangzhou 310000, Zhejiang, China

**Keywords:** cervical cancer, CSCC, ADC, immune heterogeneity, immunotherapy

## Abstract

Despite the differences in disease outcomes and pathological features between cervical squamous cell carcinoma (CSCC) and adenocarcinoma (ADC), the molecular characteristics in immune heterogeneity of the tumor microenvironment remain unclear. Here, we explored the immune landscape and heterogeneity between CSCC and ADC. Gene expression and clinical characteristics of cervical carcinoma from The Cancer Genome Atlas (TCGA) were downloaded. Differentially expressed genes (DEGs), immune cell infiltration, and pathway enrichment analyses were used to explore the immune landscape and heterogeneity between CSCC and ADC. Furthermore, distinct immune signatures between CSCC and ADC were validated based on clinical samples. In total, 4,132 upregulated DEGs and 2,307 down-regulated DEGs were identified between CSCC and ADC, with enrichments in immune related-pathways in CSCC. In addition, 54 hub DEGs correlated with patients’ prognosis and immunocytes infiltration were identified. The CSCC patients had a higher ImmuneScore and more abundant immunocytes infiltration compared to ADC patients, as validated by immunohistochemistry (IHC) and multicolor immunofluorescence (mIF) analyses of collected samples. Furthermore, CSCC displayed higher inhibitory immune checkpoints expression, tumor mutation burden (TMB), and microsatellite instability (MSI) compared to ADC, which indicated CSCC patients were more likely to benefit from immunotherapy. In summary, our results revealed the huge immune heterogeneity between CSCC and ADC, and provided guidance for immunotherapy selection for different pathological types of cervical cancer.

## INTRODUCTION

Cervical cancer is a common gynecological malignancy, which accounts for 604,127 new cases and 341,831 deaths worldwide each year [1]. The prevention and treatment of cervical cancer remains a huge burden for many developing counties. Cervical squamous cell carcinoma (CSCC) and adenocarcinoma (ADC) are two pathological types of cervical cancer, accounting for 75-90% and 10-25%, respectively [2]. The incidence and mortality of CSCC have declined sharply in recent years with the popularity of human papillomavirus (HPV) vaccines and early screening in developed countries [3]. In contrast, the proportion of ADC has gradually increased [4, 5]. Previous clinical trials contained few ADC cases, and thus the prognostic differences between the two pathological types remain to be explored.

CSCC and ADC display huge differences in the disease origin, epidemiology, molecular characteristics, tumor immune microenvironment, population distribution, pathogenic factors, clinical characteristics and prognosis [6–10]. For example, CSCC originates from the cervical squamous epithelium, while ADC originates from the endocervical glandular epithelium, which can lead to false negatives during cytology screening for early ADC [11–13]. HPV infection patterns and sensitivity to radiotherapy between CSCC and ADC are not the same, with HPV-18 infection accounting for approximately 50-58% of ADC but only 15-18% of CSCC [14–17]. Smoking is a risk factor for the carcinogenesis of CSCC, but it seems to be less correlated with ADC. On the contrary, adenocarcinoma is more closely related to other risk factors of endometrial cancer, such as miscarriage and obesity [9]. Researchers have previously identified some DEGs between CSCC and ADC, such as KRT17, IGFBP2, TRY2, CEACAM5, TACSTD1, etc. [6, 18]. Previous studies shown that adding bevacizumab to chemotherapy regimens can improve prognosis in metastatic or recurrent CSCC, but no benefits have been observed from bevacizumab in ADC [7]. In addition, compared to CSCC, ADC patients are prone to lymphatic and hematogenous metastasis, even in early stage, and the 5-year overall survival rate of ADC reduced by 10%-20% [7, 19–21]. These disease distinctions may result in different survival outcomes. Therefore, it is important to explore suitable therapeutic strategies for ADC patients.

Immunotherapy is a novel treatment for locally advanced and metastatic cervical cancer, although some patients have shown low response rates to immune checkpoint inhibitors in clinical trials [2, 22]. Immune checkpoints, tumor mutation burden (TMB), and microsatellite instability (MSI) are three major indicators for predicting the effects of immunotherapy [23, 24]. At present, it is unclear whether immunotherapy has the same therapeutic effect on CSCC and ADC, and no studies have explored immune heterogeneity between CSCC and ADC.

Here, we explored the differences in tumor immune microenvironment between CSCC and ADC to provide guidance for the selection of immunotherapy for different pathological types of cervical cancer.

## RESULTS

### Identification of DEGs between CSCC and ADC from TCGA cohorts

**Figure 1:**
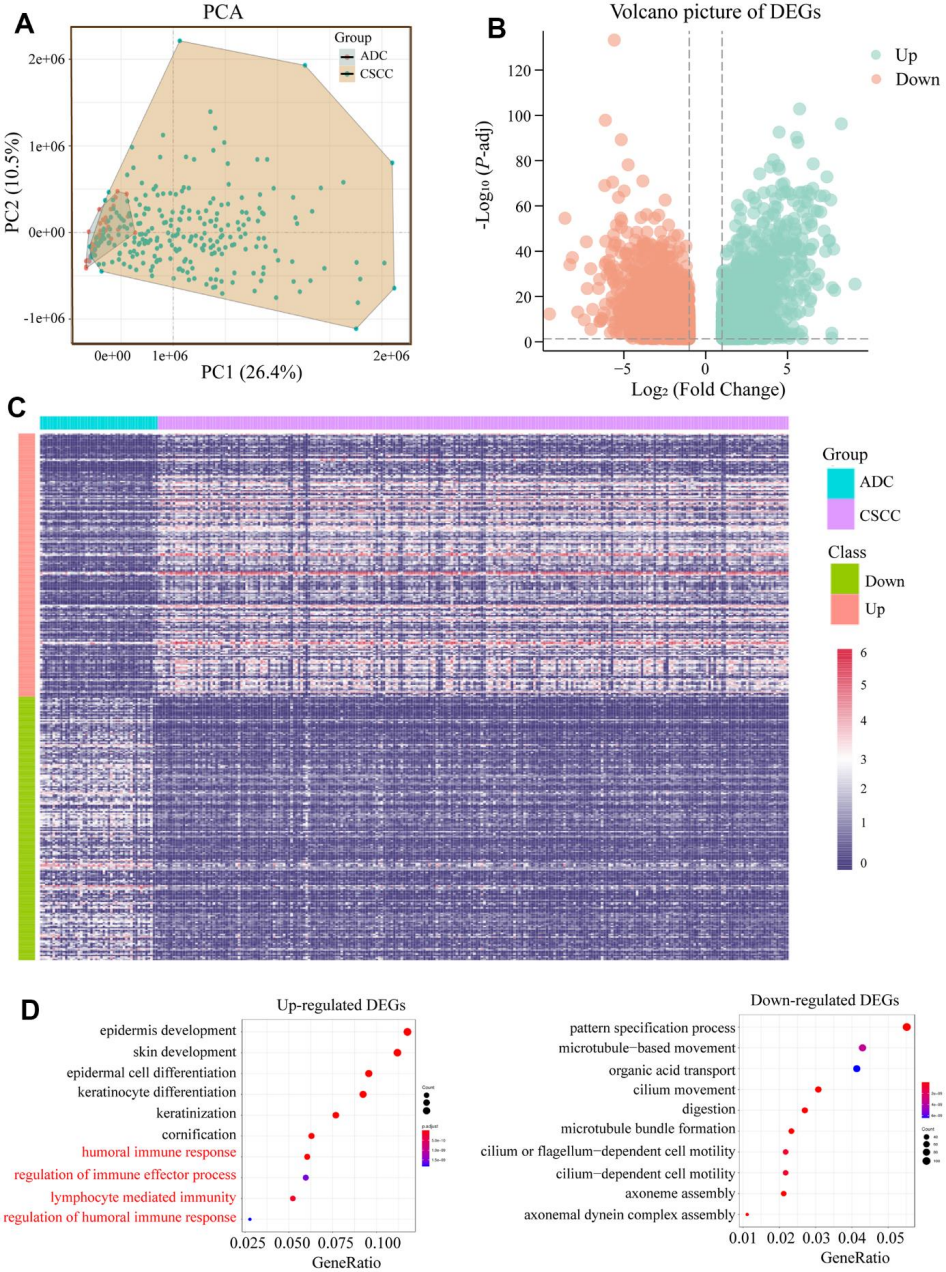
**Functional enrichment analysis of identified DEGs between CSCC and ADC.** (**A**) PCA analysis of CSCC and ADC according to their expression profiles. (**B**) Volcano picture of DEGs between CSCC and ADC. (**C**) Heatmap of the top 200 up-regulated and down-regulated DEGs. (**D**) Functional enrichment analyses of up-regulated and down-regulated DEGs, respectively. DEGs: differentially expressed genes; PCA: principal component analysis.

In total, 252 CSCC patients and 47 ADC patients were included in the study after matching gene expression and up-dated prognosis data, respectively. Detailed information is provided in Supplementary Tables 1, 2. Cervical adeno-squamous carcinoma was excluded as it is not the same as either CSCC and ADC [25–27]. We first performed principal component analysis (PCA) using the downloaded RNA-sequencing (RNA-seq) data of the CSCC and ADC cohorts. As shown in Figure 1A, the CSCC and ADC cohorts could be well distinguished, indicating different expression profiles. In total, 4 132 upregulated and 2 307 downregulated DEGs between CSCC and ADC were identified using the R package DESeq2 (LogFC >1, *P*-adj *<* 0.05) (Figure 1B and Supplementary Table 3). A heatmap was generated to display the expression profiles of the top 200 up-regulated and down-regulated DEGs in CSCC and ADC (Figure 1C). Further enrichment analysis of the DEGs revealed that CSCC patients had more immune-related activities (e.g., humoral immune response, regulation of immune effector process, lymphocyte mediated immunity, and regulation of humoral immune response) compared to ADC (Figure 1D). Gene Set Enrichment Analysis revealed that the REACTOME_SIGNALING_BY_INTERLEUKINS (NES = 2.52, *P*-adj = 0.02), REACTOME_CYTOKINE_SIGNALING_IN_IMMUNE_SYSTEM (NES = 2.36, *P*-adj = 0.02), REACTOME_NEUTROPHIL_DEGRANULATION (NES = 1.9, *P*-adj = 0.04), REACTOME_INNATE_IMMUNE_SYSTEM (NES = 1.8, *P*-adj = 0.03) were activated (Table 1).

**Table 1:** Gene set enrichment analysis of the DEGs between CSCC and ADC.

**Description**	**Enrichment score**	**NES**	**p.adjust**
REACTOME_FORMATION_OF_THE_CORNIFIED_ENVELOPE	0.74	5.05	0.02
REACTOME_KERATINIZATION	0.72	4.94	0.02
REACTOME_DEVELOPMENTAL_BIOLOGY	0.53	4.14	0.02
WP_HAIR_FOLLICLE_DEVELOPMENT_CYTODIFFERENTIATION_PART_3_OF_3	0.57	2.59	0.02
**REACTOME_SIGNALING_BY_INTERLEUKINS**	0.55	2.52	0.02
**REACTOME_CYTOKINE_SIGNALING_IN_IMMUNE_SYSTEM**	0.42	2.36	0.02
NABA_SECRETED_FACTORS	0.37	2.31	0.02
**REACTOME_NEUTROPHIL_DEGRANULATION**	0.35	1.9	0.04
**REACTOME_INNATE_IMMUNE_SYSTEM**	0.27	1.8	0.03
NABA_MATRISOME_ASSOCIATED	0.21	1.64	0.03
PID_HNF3A_PATHWAY	-0.54	-1.91	0.04
REACTOME_SLC_MEDIATED_TRANSMEMBRANE_TRANSPORT	-0.37	-2.07	0.02
WP_CILIOPATHIES	-0.49	-2.18	0.02
REACTOME_DISEASES_OF_METABOLISM	-0.53	-2.33	0.02
NABA_ECM_AFFILIATED	-0.45	-2.33	0.02
KEGG_MATURITY_ONSET_DIABETES_OF_THE_YOUNG	-0.72	-2.53	0.02
REACTOME_REGULATION_OF_BETA_CELL_DEVELOPMENT	-0.76	-2.59	0.02
PID_HNF3B_PATHWAY	-0.78	-2.75	0.02

DEGs, differentially expressed genes; CSCC, Cervical squamous cell carcinoma; ADC, adenocarcinoma.

**Figure 2:**
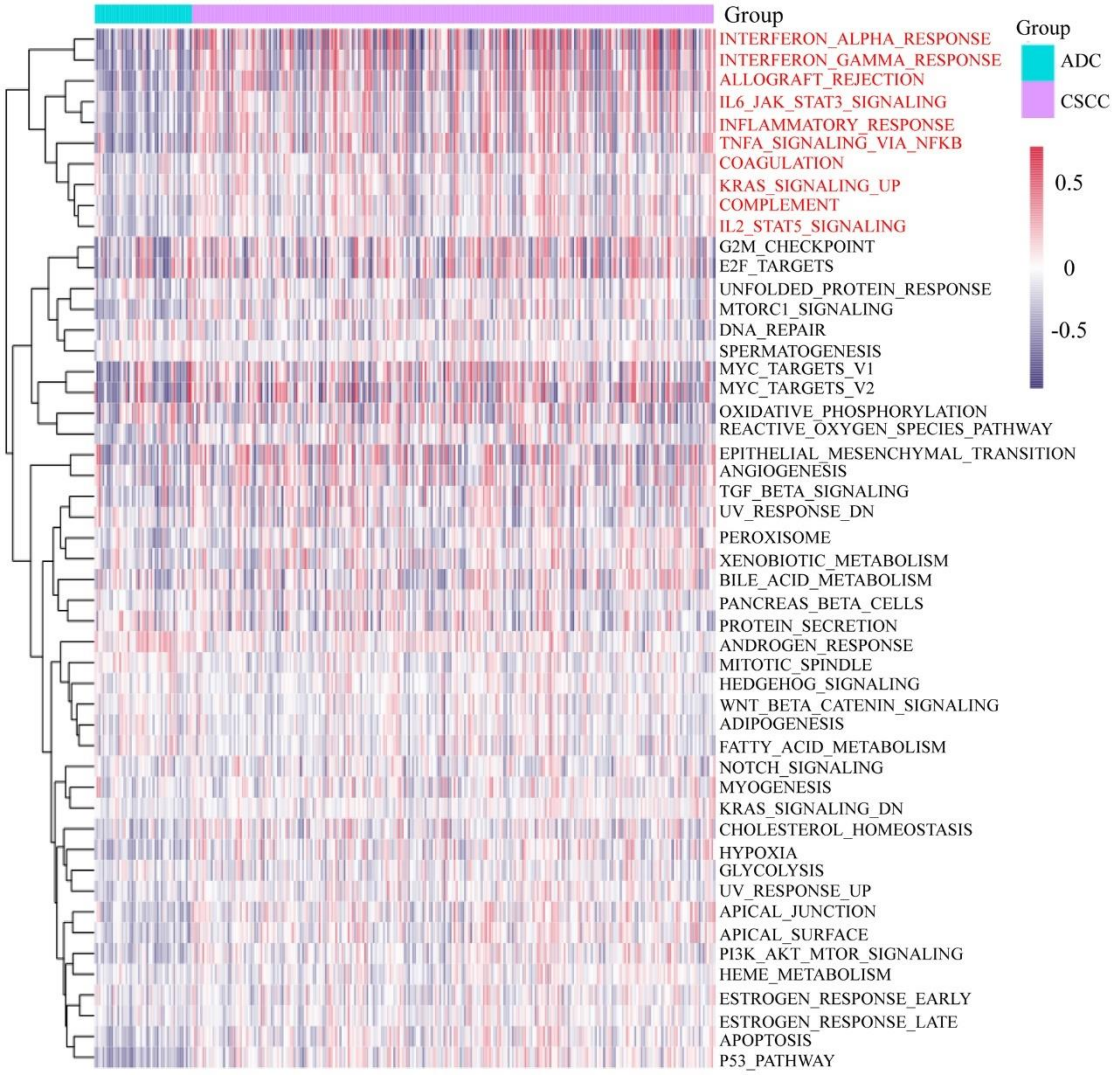
**GSVA of the 50 cancer-related signaling pathways in CSCC and ADC.** GSVA: Gene Set Variation Analysis.

Given the above findings, we next performed GSVA on CSCC and ADC patients according to the gene sets of 50 cancer related-signaling pathways. As shown in Figure 2, considerable differences were found between the CSCC and ADC cohorts. For example, CSCC patients showed higher activity in the P53 pathway, apoptosis, PI3K-AKT-MTOR-siganling, hypoxia, etc. Furthermore, immune related pathways (interferon-alpha-response, interferon-gamma-response, inflammatory response, IL6-JAK-STAT3-siganaling, complement) were also more active in CSCC patients than in ADC patients.

### Identification of pivotal IRGs and their effects on immunocytes infiltration

**Figure 3:**
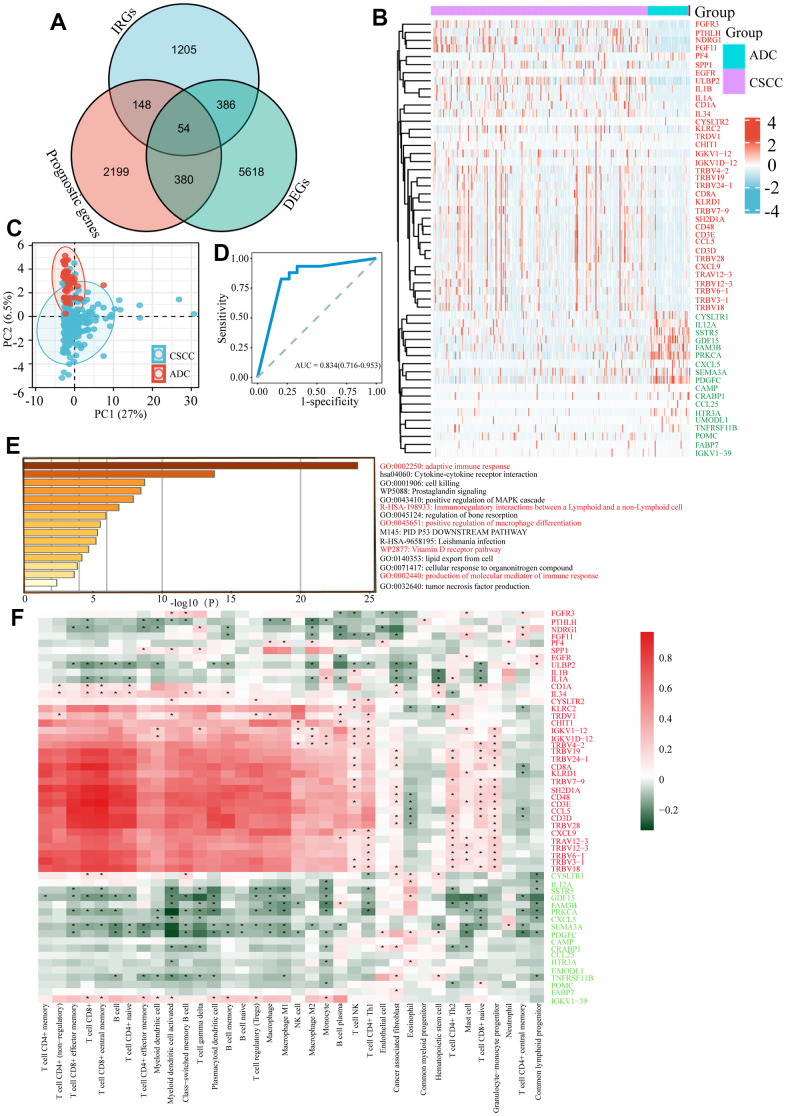
**Identification of differentially expressed IRGs between CSCC and ADC.** (**A**) Identification of 54 differentially expressed IRGs affecting patients’ prognosis between CSCC and ADC. IRGs refers to the gene list of immune related genes from the online ImmPort database. DEGs refers to the differentially expressed genes between CSCC and ADC. Prognostic genes refer to the genes affecting patients’ prognosis in cervical cancer. (**B**) Heatmap of 54 differentially expressed IRGs in CSCC and ADC cohorts. (**C**) PCA analysis of 54 differentially expressed IRGs in cervical cancer. (**D**) Receiver operating characteristic (ROC) curve based on 54 differentially expressed IRGs in cervical cancer. (**E**) Functional enrichment analysis of these 54 differentially expressed IRGs. (**F**) Relationships between different immunocytes infiltration abundance and 54 IRGs expression level. IRGs: immune related genes; PCA: principal component analysis. * *P <0.05*.

As we found that the immune related activities between CSCC and ADC patients varied greatly, we next identified key IRGs resulting in the differences. We first identified 2 781 OS related genes (namely prognostic genes) in cervical cancer patients, and obtained 1 793 IRGs from the online ImmPort database. Combined with DEGs of CSCC versus ADC, 54 differentially expressed IRGs affecting patients’ prognosis in cervical cancer were mapped using Venn diagrams (Figure 3A). Among them, 36 and 18 IRGs were up-regulated and down-regulated in CSCC versus ADC, respectively (Figure 3B). PCA based on 54 IRGs revealed some overlap between CSCC and ADC individuals (Figure 3C). We further performed ROC curve based on the 54 differentially expressed IRGs, results indicated that this gene set obtained good performance in distinguishing CSCC from ADC (AUC = 0.834 (0.716-0.953)) (Figure 3D). Functional enrichment analysis indicated that the 54 IRGs were mainly related to immune response regulation (e.g., adaptive immune response, positive regulation of macrophage differentiation, Vitamin D receptor pathway) (Figure 3E). We further explored the relationships between IRGs expression and immunocytes infiltration in cervical cancer. Interestingly, the 36 up-regulated IRGs were positively correlated with immunocytes infiltration, while the 18 down-regulated IRGs were negatively correlated with immunocytes infiltration (Figure 3F). Thus, the 54 IRGs may play pivotal roles in mediating differential immune responses in CSCC and ADC.

As the treatment options for CSCC and ADC patients do not differ significantly in clinical practice, we further identified potential small molecule drugs based on their different expression profiles using the L1000FWD database. As shown in Supplementary Figure 1A, the top ten small molecule drugs were identified based on their similarity scores. Their two-dimensional and three-dimensional architectures were further explored using the PubChem (Supplementary Figure 1B, 1C). These identified small molecule drugs could provide new insights into the treatments of different pathological cervical cancer.

### Assessment of immune heterogeneity between CSCC and ADC cohorts

**Figure 4:**
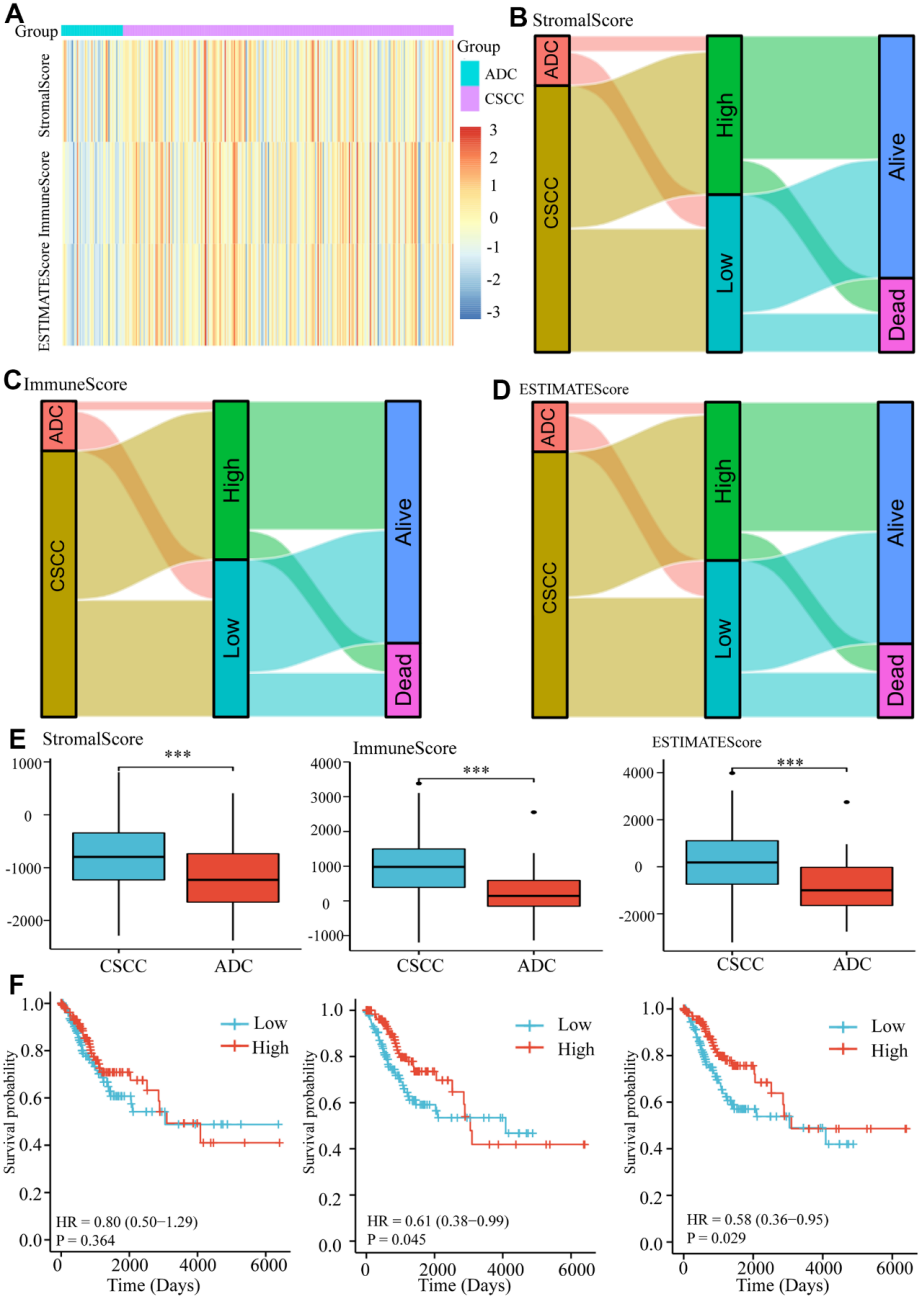
**Measurement of microenvironment scores of CSCC and ADC cohorts.** (**A**) The estimated StromalScore, ImmuneScore, and ESTIMATEScore of each cervical patient. (**B**) The Sankey diagram represents the relationships between pathological types, StromalScore and survival outcomes in cervical patients. (**C**) The Sankey diagram represents the relationships between pathological types, ImmuneScore and survival outcomes in cervical patients. (**D**) The Sankey diagram represents the relationships between pathological types, ESTIMATEScore, and survival outcomes in cervical patients. (**E**) The StromalScore, ImmuneScore, and ESTIMATEScore of CSCC and ADC cohorts. (**F**) The impacts of different microenvironment scores on survival in cervical cancer. *** *P <0.001*.

Given the diverse expression profiles of key IRGs described above, we further explored differences in microenvironment scores between the CSCC and ADC cohorts. The StromalScore, ImmuneScore, and ESTIMATEScore values of patients were first measured (Figure 4A), and Sankey diagrams were used to display the relationships between pathological types, different scores, and survival outcomes (Figure 4B–4D). Analysis revealed that CSCC patients achieved significantly higher StromalScore, ImmuneScore and ESTIMATEScore values compared to the ADC cohort (Figure 4E), which was consistent with our above observations. Furthermore, we found that higher ImmuneScore (HR = 0.61, 95%CI = 0.38-0.99, *P =* 0.045) and ESTIMATEScore (HR = 0.58, 95%CI = 0.36-0.95, *P =* 0.029) predicted better OS in cervical cancer (Figure 4F).

**Figure 5:**
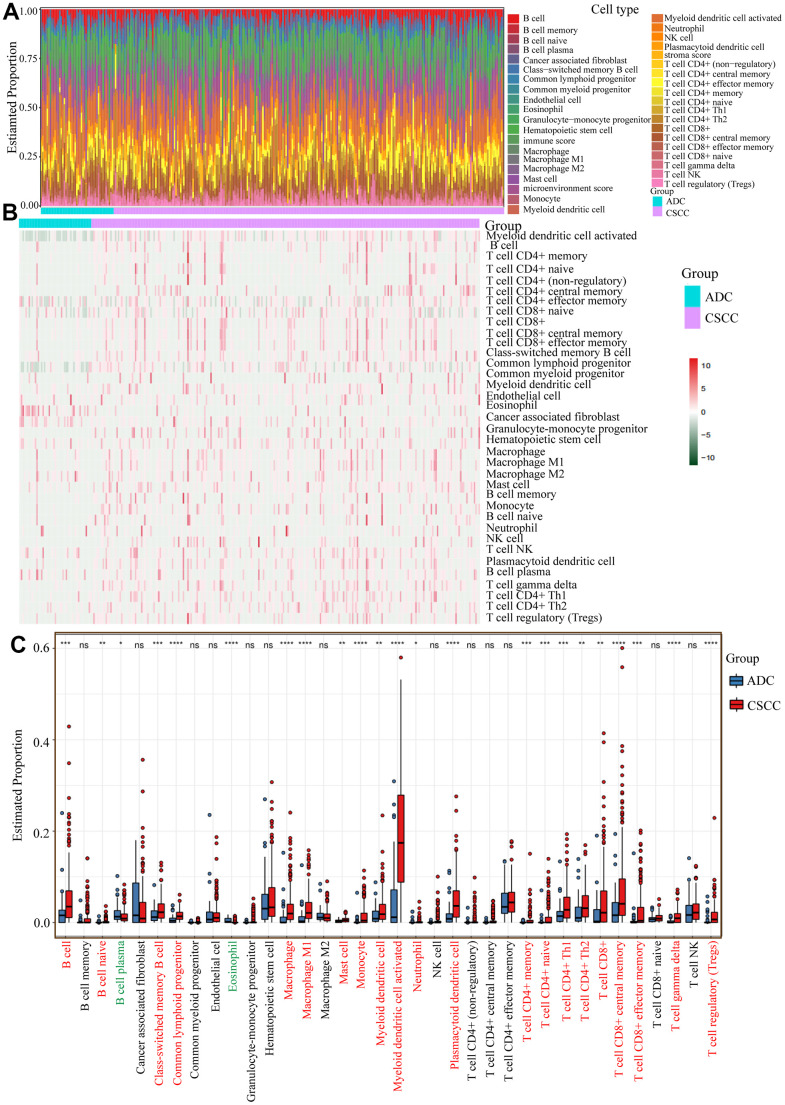
**Infiltration levels of specific immunocytes in CSCC and ADC.** (**A**) The estimated proportion of different immunocytes in cervical cancer. (**B**) The heatmap of different immunocytes distribution abundance in CSCC and ADC cohorts. (**C**) The relative estimated proportions of different immunocytes between CSCC and ADC patients. * *P* <0.05; ** < *P* <0.01; *** *P* <0.001; **** *P* <0.0001; ns: not significant.

**Figure 6:**
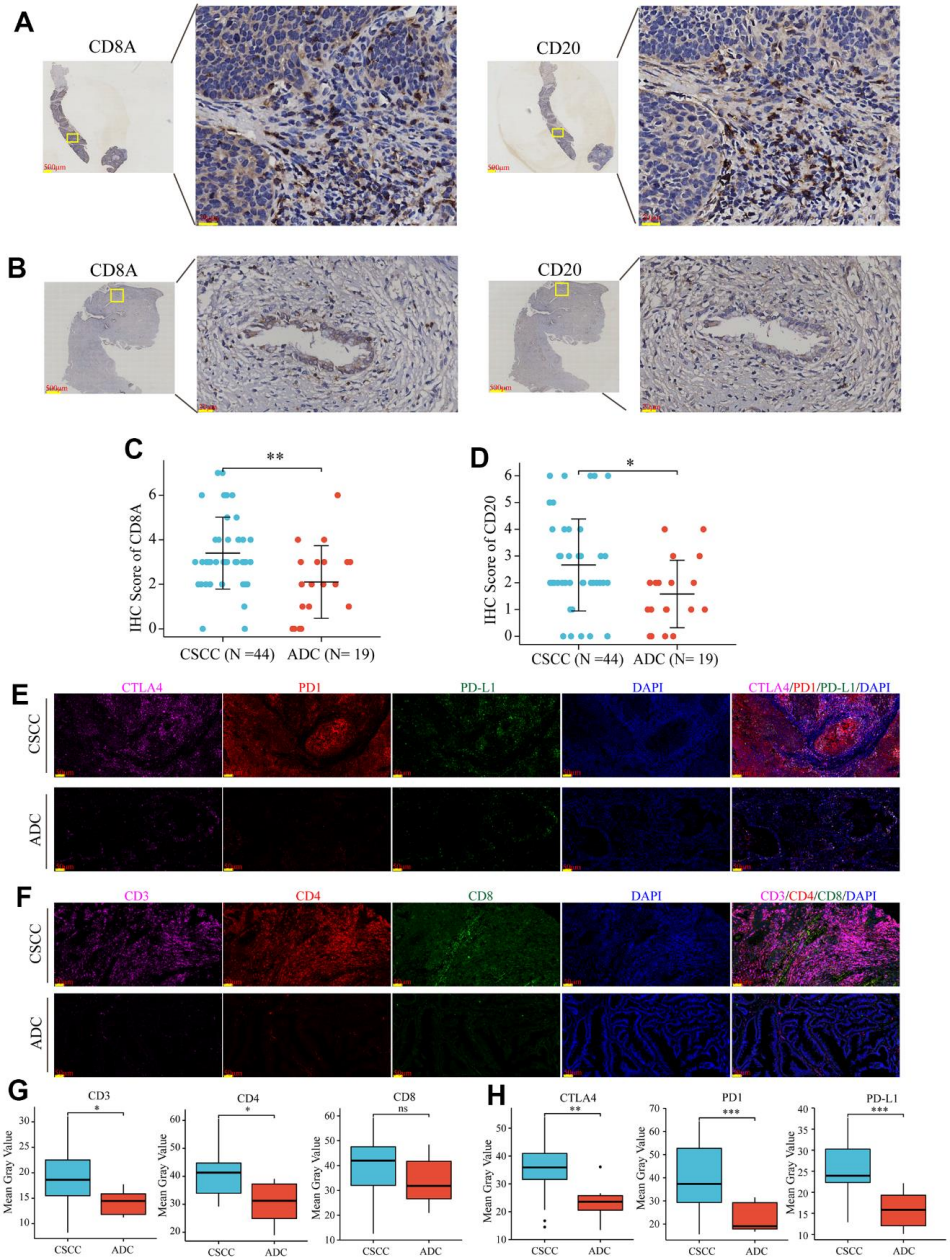
**Verification of the levels of immunocytes infiltration and inhibitory immune checkpoints expression in collected CSCC and ADC samples.** (**A**) Representative images of CD8A (T cells) and CD20 (B cells) in collected CSCC patients. (**B**) Representative images of CD8A (T cells) and CD20 (B cells) in collected ADC patients. (**C**, **D**) Statical charts displayed that CD8A (T cells) and CD20 (B cells) were more abundant in CSCC compared to ADC patients in our collected samples. (**E**, **F**) Representative staining of T cells (CD3/CD4/CD8) and inhibitory immune checkpoints (PD1/PD-L1/CTLA-4) in CSCC and ADC patients, respectively. (**G**, **H**) Statical charts displayed that T cells (CD3/CD4) and some immune inhibitory checkpoints (PD1/PD-L1/CTLA4) were more abundant in CSCC compared to ADC patients in our collected samples. * *P <0.05*; ** < *P <0.01*, *** *P* <0.001; ns: not significant.

Based on the above findings, we further explored the differences in the distribution of specific immunocytes in CSCC and ADC. As shown in Figure 5A, we measured the proportion of different immunocytes in each cervical cancer patient based on the RNA expression profiles. The heatmap showed that immunocyte abundance differed between CSCC and ADC (Figure 5B). Notably, most of the 36 common immunocytes (e.g., B cells, monocytes, and memory CD4+ and CD8+ T cells) were more abundant in CSCC than in ADC (Figure 5C). These findings were validated using IHC and mIF in collected cervical cancer samples. CD8A and CD20 were used to identify T cells and B cells, respectively. As shown in Figure 6A–6D, the average estimated scores of CD8A and CD20 were higher in 44 CSCC samples than in 19 ADC samples. We also provided the negative IHC stainings in CSCC and ADC samples as reference in Supplementary Figure 2. We then randomly selected some CSCC and ADC specimens to perform mIF staining, and results also indicated that T cells (CD3/CD4) and some immune inhibitory checkpoints (PD1/PD-L1/CTLA4) were more abundant in CSCC compared to ADC patients (Figure 6E–6H).

**Figure 7:**
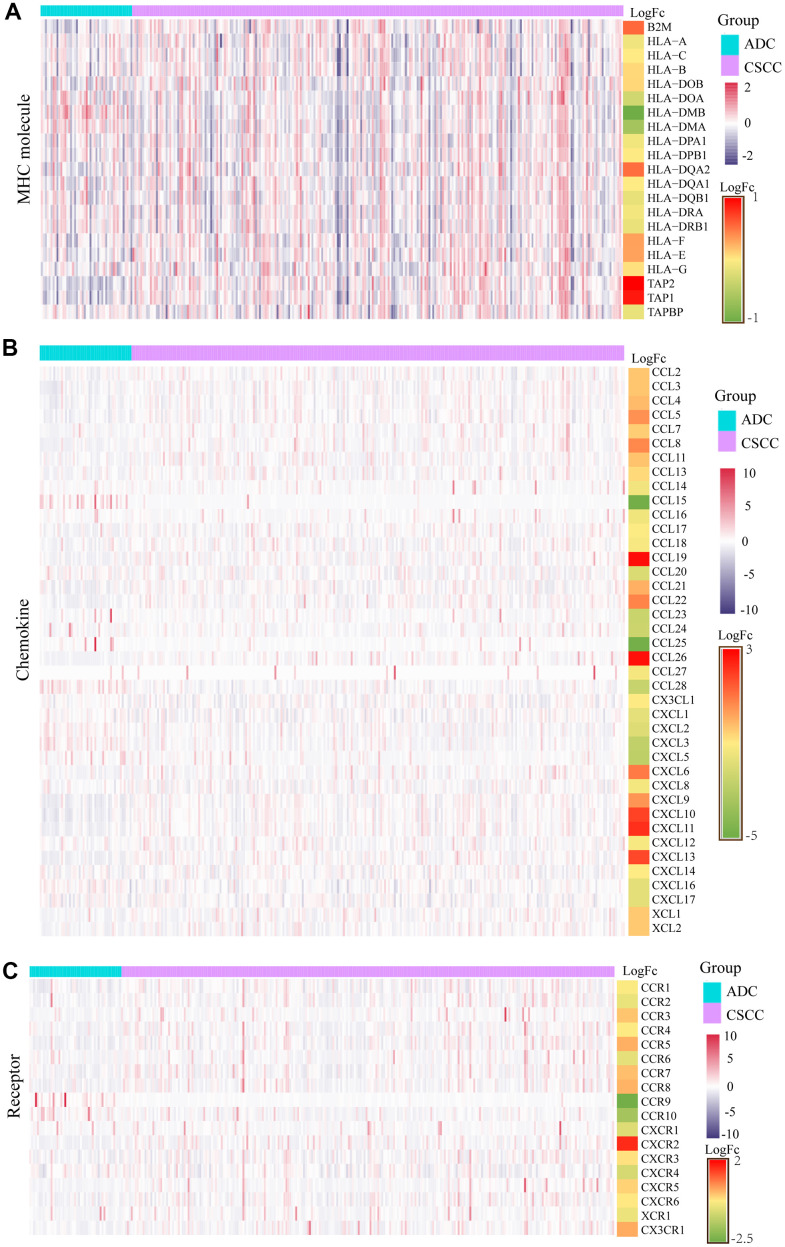
**Differential expression levels of MHC molecules, chemokines, and receptors in CSCC versus ADC.** (**A**) The differential expression of each MHC molecule in CSCC versus ADC. (**B**) The differential expression of each chemokine in CSCC versus ADC. (**C**) The differential expression of each receptor in CSCC versus ADC. MHC: major histocompatibility complex.

We also explored the expression levels of several pivotal molecules (MHC molecules, chemokines, and receptors) that may affect immunocytes infiltration. As shown in Figure 7A–7C, compared to ADC, CSCC exhibited higher expression of most molecules, consistent with these patients showing more active immune activity and abundant immunocytes infiltration.

### CSCC patients are more likely to benefit from immunotherapy

**Figure 8:**
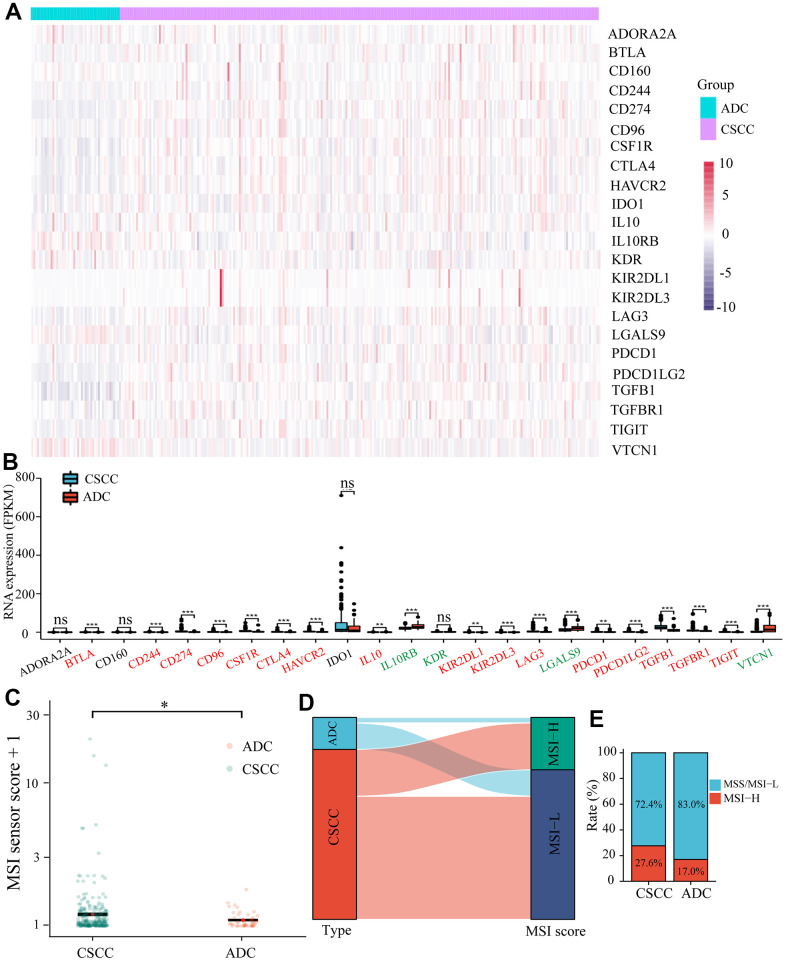
**Differences in the expression of inhibitory immune checkpoints and MSI between CSCC and ADC.** (**A**) The heatmap of different inhibitory immune checkpoints expression in each cervical cancer patient. (**B**) The expression levels of different inhibitory immune checkpoints in CSCC and ADC cohorts. (**C**) The estimated scores of MSI in CSCC and ADC cohorts. (**D**) The Sankey diagram represents the relationship between pathological type and MSI score. (**E**) The Histogram displays the proportions of different MSI in CSCC and ADC cohorts. MSI: microsatellite instability. * *P <0.05*; ** < *P <0.01*; *** *P <0.001*.

We next predicted the differences in response to immunotherapy in the two different pathological types of cervical cancer. Firstly, as shown in Figure 8A, 8B, we measured the expression levels of 23 common inhibitory immune checkpoints in CSCC and ADC. In total, 16 (*CD274, CTLA4*, *BTLA*, *CD244*, *CD96*, *CSF1R*, *HAVCR2*, *IL10*, *KIR2DL1*, *KIR2DL3*, *LAG3*, *PDCD1*, *PDCD1LG2*, *TGFB1*, *TGFBR1*, and *TIGIT*) and four (*IL10RB*, *KDR*, *LGALS9*, and *VTCN1*) were significantly up-regulated and down-regulated in CSCC versus ADC, respectively.

**Figure 9:**
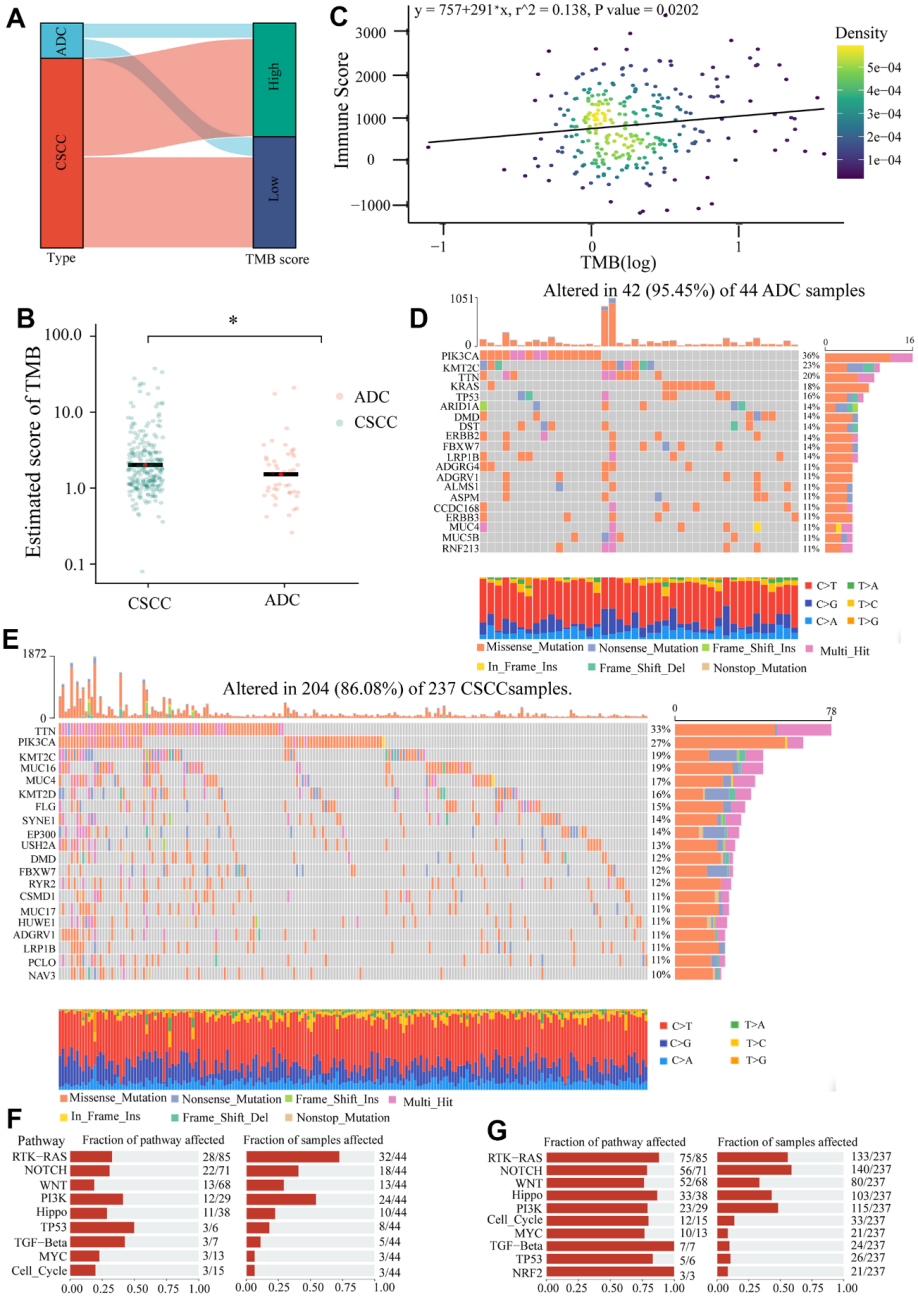
**The differences of TMB and gene mutation between CSCC and ADC patients.** (**A**) The Sankey diagram represents the relationship between pathological type and TMB score. (**B**) The estimated score of TMB in CSCC and ADC. (**C**) The relationship between ImmunoScore and TMB score. (**D**) The top 20 mutated genes in 42 of 44 ADC samples. (**E**) The top 20 mutated genes in 204 of 237 CSCC samples. (**F**) The oncogenic pathways of mutated genes and their corresponding fractions of samples affected in ADC samples. (**G**) The oncogenic pathways of mutated genes and their corresponding fractions of samples affected in CSCC samples. * *P <0.05*.

Secondly, we measured the differences in MSI between SCC and ADC. The MSI score was significantly higher in CSCC than in ADC (Figure 8C). The Sankey diagram in Figure 8D represented the relationship between pathological type and MSI score. The proportion of MSI-H in CSCC (27.6%) was much higher compared to that in ADC (17.0%) (Figure 8E). Previous studies have shown that TMB could affect immune response by introducing neoantigens. The Sankey diagram in Figure 9A, 9B displayed the relationship between TMB and pathological type in cervical cancer. Results indicated that the TMB score was higher in CSCC than in ADC. Further analysis revealed that ImmuneScore was positively correlated with TMB score in cervical cancer (r^2^ = 0.138, *P =* 0.0202) (Figure 9C). We also explored the gene mutation characteristics of CSCC and ADC. As shown in Figure 9D, 9E, the top 20 altered genes and their mutation frequencies were not identical in CSCC and ADC. *PI3KCA*, *KMT2C*, *TTN*, *DMD*, *FBXW7*, *LRPIB*, *ADGRV1*, and *MUC4* were shared by the CSCC and ADC cohorts. The predominant pathways affected and their corresponding scores also varied widely between the affected CSCC and ADC cohort samples (Figure 9F, 9G). For example, the top 1 affected pathway was RTK-RAS, and the affected rate in ADC (32/44) was much higher than CSCC (133/237) group. Thus, compared to ADC, the CSCC cohort displayed higher inhibitory immune checkpoints expression, TMB, and MSI. Therefore, we speculated that CSCC patients are more likely to benefit from immunotherapy.

## DISCUSSION

Over the past several decades, the epidemiological characteristics of CSCC and ADC have undergone great changes. The proportion of ADC is increasing year on year, especially in young patients [5, 28, 29]. Indeed, there are substantial differences between CSCC and ADC regarding genomic transcription, radiotherapy sensitivity, and lymphatic metastasis probability [7, 19, 30, 31]. However, there are no differences in traditional therapeutic strategies for CSCC and ADC patients [17, 32]. Therefore, by exploring the heterogeneity between these two different pathological types, specific treatments for ADC may be developed [33].

A greater abundance of tumor-infiltrating lymphocytes tends to improve the response rate and efficacy of immunotherapy [34]. The current study is the first to explore the differences in immune microenvironment between CSCC and ADC using public databases and collected cervical cancer patients. Results showed that immune-related signaling pathways were more active in CSCC than in ADC patients. Infiltration levels of 21 (21/36) common immunocytes (e.g., B cells, macrophage M1, CD4+ and CD8+ T cells) and estimated ImmuneScores were significantly higher in the CSCC cohort than in the ADC patients. Furthermore, several immune-related molecules (inhibitory checkpoints, MHC molecules, chemokines, and receptors) were more abundant in CSCC than in ADC. Thus, these findings indicate that CSCC patients exhibit more abundant immunocytes infiltration and may be more susceptible to immunotherapy.

Immune checkpoint inhibitors (e.g., PD-1/PD-L1, CTLA-4) have shown potential therapeutic effects in cervical cancer [35–37]. However, only a small proportion of cervical cancer patients benefit from immunotherapy due to a low overall response rate [38–40]. Therefore, there is an urgent need to explore new strategies to improve the response rate of immunotherapy. Previous studies have identified potential marker genes (*CSF1R*, *ERAP1*, *LDHA*, etc.,) that may affect response rate by reshaping the cervical cancer microenvironment [40–42]. Yang et al. identified 11 IRGs associated with patient prognosis and response rate to immunotherapy in cervical cancer [43]. Recent years, some single-cell data of cervical cancer has been published, which may provide a better means to explore the immune microenvironment of different pathological types of cervical cancer [44, 45]. For example, Hua et al. performed single-cell RNA sequencing on 3 CSCC and 5 ADC samples, and revealed their heterogeneity in immunocytes infiltration [44]. Yao et al. also constructed an immune cell infiltration scoring system containing IL1B, CST7, and ITGA5 to predict the efficacy of immunotherapy for cervical cancer [46]. Here, we also identified 54 differentially expressed IRGs between CSCC and ADC, which may be promising targets for exploring immune heterogeneity between different pathological types of cervical cancer and improving the low response rate of immunotherapy in cervical cancer.

Generally, TMB refers to the number of somatic non-synonymous mutations or all mutations per megabase in the gene region detected by whole-exome sequencing or targeted sequencing of a tumor sample (tumor tissue or peripheral blood) [47]. It can indirectly reflect the ability and extent of tumors to produce new antigens, which can activate stronger immune responses through MHC presentation [48]. In addition, TMB is an indicator for predicting the efficacy of immunotherapy in various types of malignancies [23, 49–53]. Previous studies have confirmed that higher TMB was positively associated with immunocytes infiltration in ovarian and gastric cancer [54, 55]. In our study, the TMB score was positively correlated with the ImmuneScore, and was higher in the CSCC cohort than in the ADC cohort. These findings suggest that TMB may facilitate immunocytes infiltration in cervical cancer, and CSCC patients are more likely to benefit from immunotherapy.

Low response rate of cervical cancer patients to immunotherapy remains a major challenge for clinicals, and reliable indicators for immunotherapy should be developed. Nowadays, MSI, TMB and inhibitory checkpoints expression are the commonly used indicators to evaluate the efficiency of immunotherapy, and patients with higher TMB, MSI or inhibitory checkpoints expression are more likely to benefit from immunotherapy [22, 23, 56]. In cervical cancer patients, we found CSCC cohorts obtained higher TMB score, MSI score and common inhibitory checkpoints expression (e.g., *PDCD1*, *CD274*, *BTLA*, *CTLA4*, *TIGIT*, etc.), accompanied by more abundant immunocytes infiltration. These findings indicate CSCC patients are more likely to benefit from immunotherapy by activating exhausted immunocytes and remodeling the immunosuppressive tumor microenvironment. Our study may also provide a new perspective for personalized immunotherapy based on patients’ pathological type.

We note several limitations in our study. First, previous studies have found differences in HPV infection patterns between CSCC and ADC, which may lead to different immune microenvironments [14–16]. Therefore, differences in HPV infection and integration between CSCC and ADC should be explored in the future. Second, the number of collected clinical cervical cancer patients was limited to 44 CSCC and 19 ADC patients. Collecting and analyzing more cases will help verify the findings obtained from the public database. Finally, cellular and animal models of CSCC and ADC are also needed to further explore their immune heterogeneity.

## MATERIALS AND METHODS

### Data sources

The expression profiles (FPKM, Counts) of cervical cancer patients were downloaded from the TCGA (https://portal.gdc.cancer.gov/). The updated prognostic information of cervical cancer patients was obtained from a data resource [57]. The somatic mutation data of cervical cancer patients were downloaded from TCGA database by the R packages TCGAbiolinks (v 2.18.0) [58]. The list of immune related genes (IRGs) was obtained from the online ImmPort (https://www.immport.org/shared/genelists).

### Identification of DEGs between CSCC and ADC

DEGs between CSCC and ADC were identified using the R package DESeq2 based on HTSeq-counts data [59]. The selection criteria were |log2 (foldchange)| > 1 and *P*-adj < 0.05.

### Gene set variation analysis (GSVA) analysis

The hallmark gene sets of the 50 cancer-related signaling pathways were obtained from the online MSigDB (v.7.4) (https://www.gsea-msigdb.org/gsea/msigdb/). GSVA R package was used to calculate the standardize enrichment score of the related signaling pathway [60].

### Functional enrichment analysis

The functional enrichment analysis and Gene Set Enrichment Analysis of DEGs between CSCC and ADC in our studies was performed by the online Metascape (https://metascape.org/gp/index.html#/main/step1) and clusterProfiler R package, respectively [61–63].

### Immune analysis by ESTIMATE and xCell package

We used the gene expression profiles to measure the microenvironment scores of each cervical cancer patient by the ESTIMATE package [64]. The estimated proportion of 36 distinct immunocytes in each cervical patient was measured by the xCell package (https://github.com/dviraran/xCell) [65].

### Survival analysis

299 cervical cancer patients were divided into high or low group based on the median microenvironment score. Survival curves were drawn using the “survival” and “survminer” R package. 2 781 pivotal genes affected patients’ overall survival (OS) were also identified by the “survival” and “survminer” R package.

### Measurement of TMB for cervical cancer patients

We used the “maftools” R package to measure the tumor mutation burden (TMB) for each patient with cervical cancer from TCGA [66].

### Measurement of MSI for cervical cancer patients

MSI is defined as genetic instability in short nucleotide repeats (microsatellites) because high mutation rates may lead to abnormal DNA mismatch repair. The MSI sensor scores of cervical cancer are obtained from the previous study of Li et al. [67], which used MSIsensor to measure the MSI score of each sample in TCGA database [56]. Three-quarters as the threshold, greater than threshold is considered MSI-H, the rest is defined as MSI-L.

### Identification of potential small molecule drugs

Potential small molecule drugs were identified based on the 54 differentially expressed IRGs between CSCC and ADC patients using the L1000FWD database (L1000 Fireworks Display (maayanlab.cloud) [68]. Their two/three-dimensional architecture were further explored by the PubChem website (https://pubchem.ncbi.nlm.nih.gov/) [69].

### Receiver operating characteristic (ROC) curve

70% samples from the TCGA dataset were used as the training cohort, and the remaining 30% were used as the testing cohort. Based on the expression of 54 differentially expressed IRGs, a logistic regression model was established on the training set using “glm” R package. The “pROC” R package was used to evaluate the classification performance of the model on the testing cohort.

### IHC and mIF analysis of collected cervical cancer samples

The tissue microarray containing 44 CSCC and 19 ADC cervical cancer specimens was collected from the department of gynecologic oncology of Tongji Hospital (Ethical permission number: TJ-IRB20210609), and the detailed clinical data of 63 cervical cancer samples were provided in the Supplementary Table 4. As we described previously [70], SP kit (ZSGB BIO, #SP-9001) was used to perform IHC staining according to the manufactures’ protocol. Briefly, 4μm paraffin-embedded (FFPE) tissue slides were put into dewaxing solution I/II, 100% ethanol, 90% ethanol, 85% ethanol, and 75% ethanol for 5 minutes, respectively. Then slides were sequentially restored by antigen and blocked endogenous peroxidase with citric acid antigen retrieval buffer (PH6.0) and 3% hydrogen peroxide, respectively. The slides were incubated with primary antibodies of CD8A (A0663, 1:50, ABclonal) and CD20 (A4893, 1:50, ABclonal) at 4° C for overnight. Estimated staining score of CD8A and CD20 of each tissue slide were measured according to the positivity percentage (0-5% = 0, 6-25% = 1, 26-50% = 2, 51-75% = 3, >75% = 4) and staining intensity (negative = 0, weak = 1, moderate = 2, strong =3) by two independent professional clinicians.

The experimental procedure of mIF was basically the same as that of IHC. After incubation with the first primary antibody, sections were incubated with 488-TSA (1:4000, PINUOFEI, Wuhan) for 30 minutes. Sections were then incubated with the secondary antibody after antigen retrieval. Finally, sections were subjected to CY5-TSA (1:1000, PINUOFEI, Wuhan) for 30 minutes after antigen retrieval, and further incubated with third antibody for overnight. The nuclei were stained with DAPI (C0060; Solarbio, Beijing) for ten minutes. The details of antibodies were as follows: CD3 (ab16669, 1:150, Abcam), CD4 (RMA-0620, MXB), and CD8 (RMA-0514, MXB), PD1 (86163, 1:200, CST), PD-L1 (13684, 1:100, CST), CTLA4 (53560, 1:100, CST). All the sections were scanned by the 3D panoramic scanner (DANJIER, HISHTECH Pannoramic 250, Jinan) and further visualized by the CaseViewer.

### Statical analysis

All statical analyses were performed using the R (version 4.1.0) (https://www.r-project.org/). The Wilcoxon test was employed to compare two groups. The Pearson correlation coefficient was used to measure the relationship between Immunescore and TMB score. Statical significance was set at *P* < 0.05. * *P* <0.05; ** < *P* <0.01; *** *P* <0.001; **** *P* <0.0001; ns: not significant.

### Availability of data and materials

The expression profiles (FPKM, Counts) of cervical cancer patients were downloaded from the TCGA (https://portal.gdc.cancer.gov/). The list of immune related genes (IRGs) was obtained from the online ImmPort (https://www.immport.org/shared/genelists). The hallmark gene sets of the 50 cancer-related signaling pathways were obtained from the online MSigDB (v.7.4) (https://www.gsea-msigdb.org/gsea/msigdb/).

## Supplementary Material

Supplementary Figures

Supplementary Table 1

Supplementary Table 2

Supplementary Table 3

Supplementary Table 4
