# Supplementary material for: Immune landscape and heterogeneity of cervical squamous cell carcinoma and adenocarcinoma
Source: Aging (Albany NY). 2024 Jan 10;16(1):568–92. doi: 10.18632/aging.205397 (PMC10817369; doi:10.18632/aging.205397)
Supplement: Supplementary Figures [file aging-16-205397-s001.pdf]

SUPPLEMENTARY FIGURES

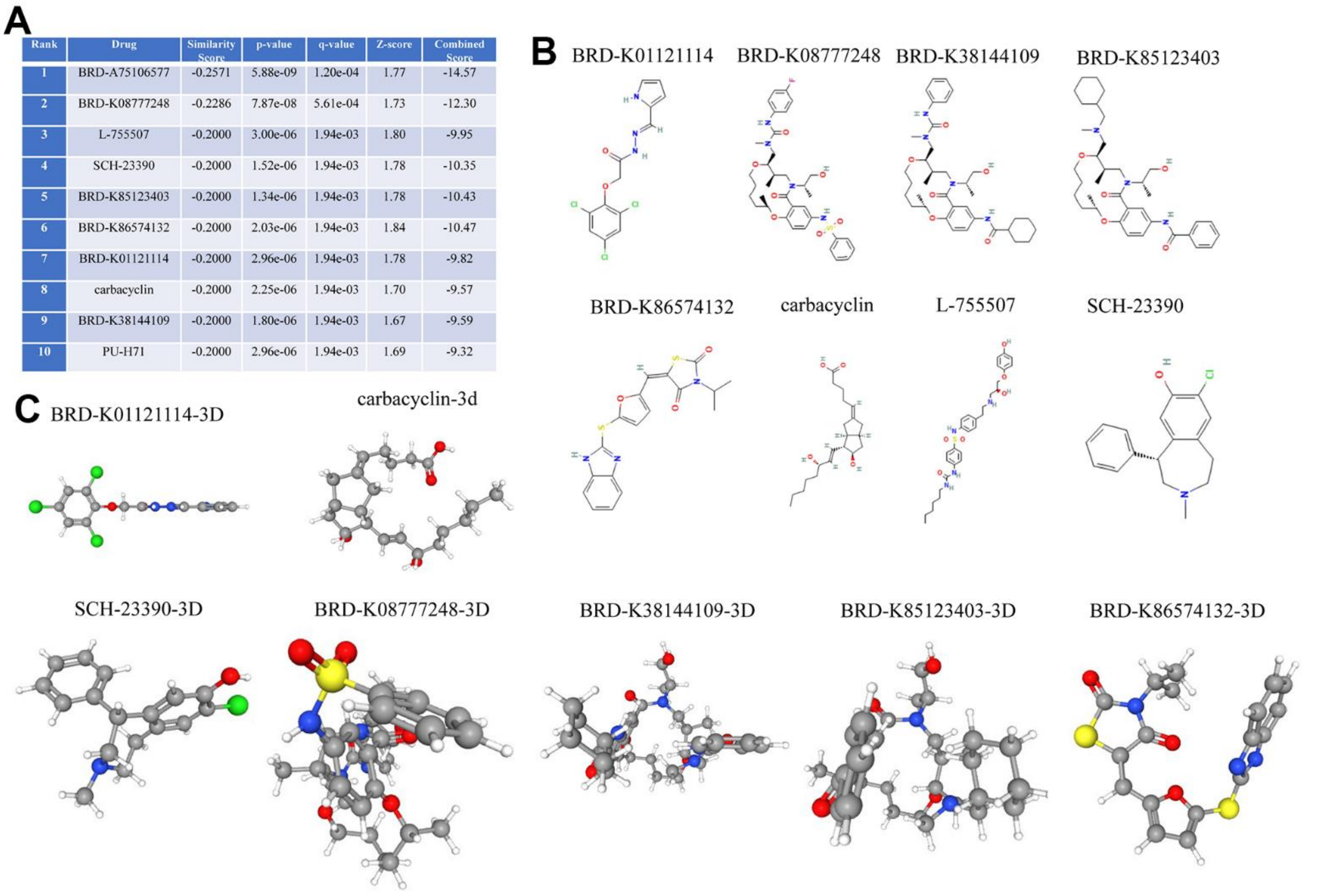

Rank	Drug	Similarity Score	p-value	q-value	Z-score	Combined Score
1	BRD-A75106577	-0.2571	5.88e-09	1.20e-04	1.77	-14.57
2	BRD-K08777248	-0.2286	7.87e-08	5.61e-04	1.73	-12.30
3	L-755507	-0.2000	3.00e-06	1.94e-03	1.80	-9.95
4	SCH-23390	-0.2000	1.52e-06	1.94e-03	1.78	-10.35
5	BRD-K85123403	-0.2000	1.34e-06	1.94e-03	1.78	-10.43
6	BRD-K86574132	-0.2000	2.03e-06	1.94e-03	1.84	-10.47
7	BRD-K01121114	-0.2000	2.96e-06	1.94e-03	1.78	-9.82
8	carbacyclin	-0.2000	2.25e-06	1.94e-03	1.70	-9.57
9	BRD-K38144109	-0.2000	1.80e-06	1.94e-03	1.67	-9.59
10	PU-H71	-0.2000	2.96e-06	1.94e-03	1.69	-9.32

Supplementary Figure 1. The characteristics of ten potential small molecule drugs identified by L1000FWD. (**A**) The ten identified small molecule drugs based on the similarity scores. (**B**) The two-dimensional architectures of some identified small molecule drugs. (**C**) The three-dimensional architectures of some identified small molecule drugs.

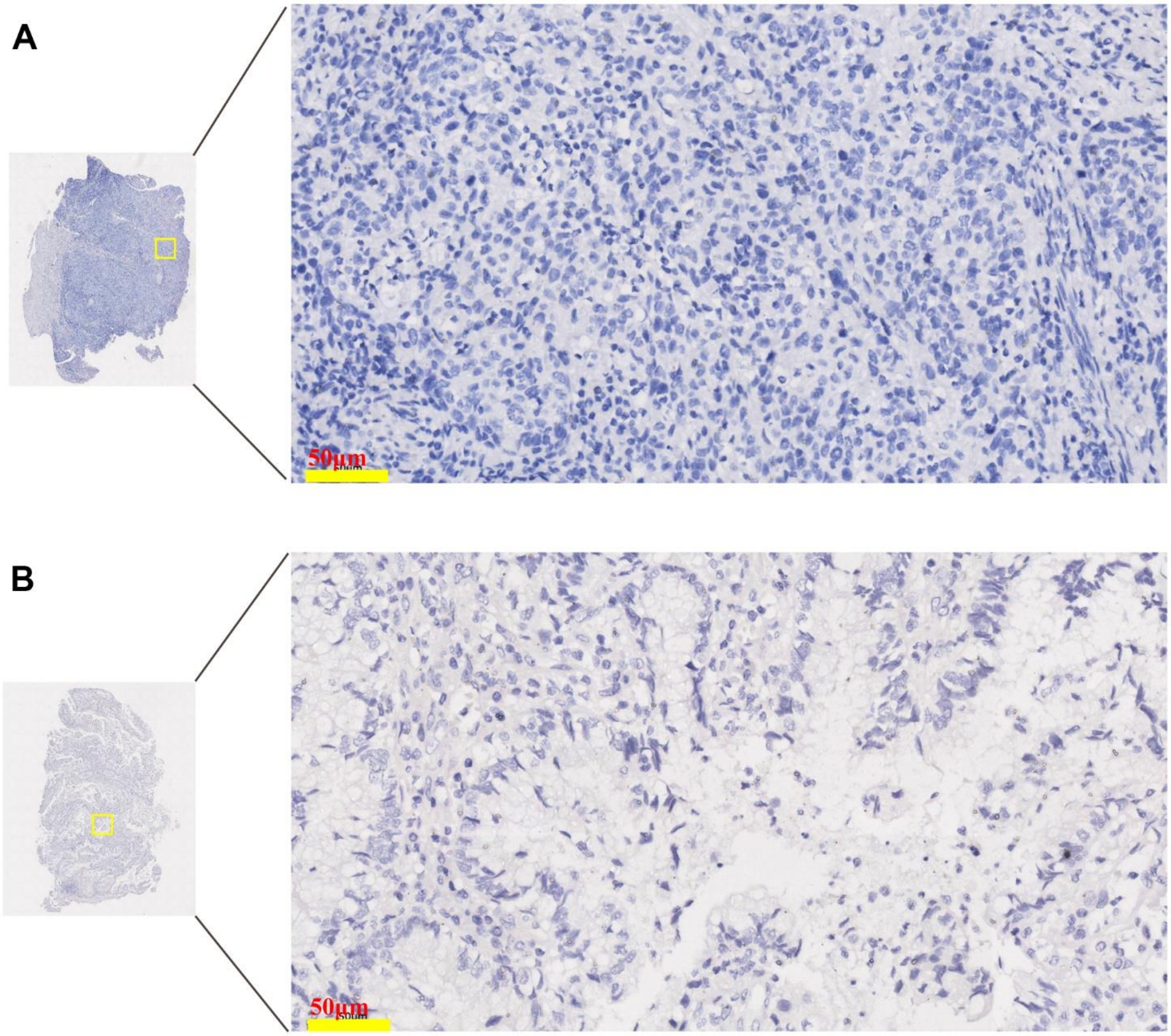

Supplementary Figure 2. Negative immunohistochemistry staining of CD8A and CD20 in collected cervical samples. (**A**) Negative immunohistochemistry staining in CSCC samples. (**B**) Negative immunohistochemistry staining in ADC samples.
