# Supplementary material for: Immune landscape and heterogeneity of cervical squamous cell carcinoma and adenocarcinoma
Source: Aging (Albany NY). 2024 Jan 10;16(1):568–92. doi: 10.18632/aging.205397 (PMC10817369; doi:10.18632/aging.205397)
Supplement: Supplementary Table 4 [file aging-16-205397-s005.pdf]

Supplementary Table 4. Characteristics of included patients with cervical cancer for tissue microarray.

ID	Pathological type	Age	Stage	Grade	Lymph node metastasis
1	ADC	42	IIIC1	G2	Yes
2	ADC	44	IB1	G2	No
3	ADC	66	IB2	G2	Yes
4	ADC	30	IB3	G2	Yes
5	ADC	49	IIIC2	G1	No
6	ADC	49	IIIC2	G1	No
7	ADC	52	IIIC1	G1	No
8	ADC	45	NA	G1	Yes
9	ADC	35	IB3	G1	No
10	ADC	45	IA	G1	No
11	ADC	55	IIA1	G1	Yes
12	ADC	36	IIIC2	G1	No
13	ADC	52	IB1	G1	No
14	ADC	42	IB3	G3	Yes
15	ADC	56	IB1	G3	No
16	ADC	63	IIIC	G3	No
17	ADC	47	IB1	G3	No
18	ADC	44	IIIB	G3	Yes
19	ADC	28	IB3	NA	No
20	CSCC	50	IB3	G2	Yes
21	CSCC	48	IIB	G2	No
22	CSCC	69	IB2	G2	No
23	CSCC	57	IIA1	G2	No
24	CSCC	51	IIIC1	G2	No
25	CSCC	51	IB1	G2	No
26	CSCC	52	IB2	G2	Yes
27	CSCC	49	IIIC1	G2	No
28	CSCC	48	IB2	G2	Yes
29	CSCC	41	IIA2	G2	Yes
30	CSCC	49	IB1	G2	No
31	CSCC	48	IIA2	G2	No
32	CSCC	43	IB3	G2	Yes
33	CSCC	55	IIIC1	G2	No
34	CSCC	43	IB3	G2	No
35	CSCC	51	IIA1	G2	No
36	CSCC	39	IB3	G2	No
37	CSCC	51	IB2	G2	No
38	CSCC	57	IIIC1	G2	No
39	CSCC	62	IIIC1	G2	No
40	CSCC	51	IB2	G2	No
41	CSCC	38	IB2	G2	No

42	CSCC	43	IB2	G2	No
43	CSCC	48	IB1	G1	No
44	CSCC	56	IB3	G1	Yes
45	CSCC	58	IIA1	G1	No
46	CSCC	30	IB3	G1	No
47	CSCC	62	IIIC1	G1	Yes
48	CSCC	51	IB3	G1	Yes
49	CSCC	37	IIIC1	G1	No
50	CSCC	44	IIA2	G1	No
51	CSCC	52	IIIC2	G1	Yes
52	CSCC	64	IIB	G1	Yes
53	CSCC	68	IIA1	G1	No
54	CSCC	55	IB2	G1	Yes
55	CSCC	28	IIIC1	G1	No
56	CSCC	70	IIB	G1	Yes
57	CSCC	55	IIIC	G1	Yes
58	CSCC	57	IIIC2	G1	No
59	CSCC	66	IIA2	G1	Yes
60	CSCC	48	IB3	G1	No
61	CSCC	58	IIA	G1	No
62	CSCC	44	IB3	G1	Yes
63	CSCC	51	IIIC1	G3	No

CSCC, Cervical Squamous Cell Carcinoma; ADC, Cervical Adenocarcinoma; NA, Not Available.
